# Sleep quality of nurses in the emergency department of public hospitals in China and its influencing factors: a cross-sectional study

**DOI:** 10.1186/s12955-020-01374-4

**Published:** 2020-04-29

**Authors:** Hongyun Dong, Qiong Zhang, Chunji Zhu, Qian Lv

**Affiliations:** 1 Shouguang People’s Hospital. Shouguang People’s Hospital, NO. 3173 Jiankang Street, Shouguang, Weifang, 262700 Shandong Province China; 2 School of Nursing, Weifang University of Science and Technology, NO. 1299 Jinguang Street, Shouguang, Weifang, 262700 Shandong Province China

**Keywords:** Sleep, Nurse, Emergency, Factor, Stress, Workload

## Abstract

**Background:**

Studies have shown that poor sleep could result in many unpleasant consequences and is prevalent in nurses. Considering the fact of high stress, overwhelming workload and many night shifts in the emergency department in China, this study aimed to evaluate the current status of emergency nurses’ sleep quality in public hospitals in Shandong, China and explored its influencing factors.

**Methods:**

A self-administered questionnaire incorporating the Job Content Questionnaire and Pittsburgh Sleep Quality Index (PSQI) was conducted among 4856 emergency nurses in five randomly selected city emergency command systems in Shandong, China. The association of potential influencing factors, including occupational, psychosocial and individual factors, with poor sleep (PSQI> 5) was quantified by multivariate logistic regression analysis.

**Results:**

The average PSQI score of 4730 emergency nurses in public hospitals was 8.2 ± 3.9, including 3114 (65.8%) subjects with PSQI > 5 and 2905 (61.4%) > 8; these figures were found highest for 337 emergency nurses in 14 tertiary hospitals with 11.8 ± 4.3, 257 (76.3%) and 232 (68.8%), followed by 1044 emergency nurses in 43 secondary hospitals with 9.5 ± 3.9, 725 (69.4%) and 675 (64.7%) and 3349 emergency nurses in 167 primary hospitals with 7.4 ± 3.5, 2132 (63.7%) and 1998 (59.7%). The following factors were associated with poor sleep: hospital level (tertiary vs. primary, secondary vs. primary), female sex, less of exercise, long work hours per week, many patients in the charge of at night, high monthly night shift frequency (4–6 vs. never, ≥7 vs. never) and high occupational stress.

**Conclusions:**

The sleep quality of emergency nurses in public hospitals in China was poor, especially in tertiary hospitals. Many factors as listed above, especially occupational stress, night shift taking and workload at night, should be considered when improving emergency nurses’ sleep quality.

## Background

With increasing modernization and accelerating life rhythms, sleep is increasingly becoming an important global public health issue influencing millions of people [1]. Poor sleep could bring about unpleasant consequences such as irritability, memory loss, inattention in the workplace, work-related accidents or injuries, absence and even depression [2–7]. Sleep restriction could lead to metabolic and endocrine disorders [8], and increased blood pressure and heart rate [9–11]. Poor sleep quality has been shown to be associated with negative health outcomes such as increased risk of obesity, heart attack, hypertension, diabetes, and stroke [9, 12–15] and might have a direct effect on high mortality [16, 17]. High risks of death, stroke, coronary heart disease and cardiovascular disease have been reported to be associated with a short sleep duration by systematic reviews or meta-analyses [18, 19]. Poor sleep could also be taken as an early sign of underlying health problems [20].

Nurses have been reported to suffer from poor sleep, especially in large-scale hospitals [21–24]. In the United States [25], of 540 nurses working in six hospitals, 418 (77.4%) had a poor quality of sleep. In Taiwan, 248 (57.0%) of 435 female nurses reported poor sleep [26] and in Norway, 32.4–37.6% of 1968 nurses experienced symptoms of sleepiness and insomnia [27]. In China, the prevalence of poor sleep was reported to be 63.5% among 4951 nurses in tertiary hospitals [23] and severe sleep problems 54.8% [24]. Nurses in emergency department undertake tasks of pre-hospital first aid, emergency patient observation and rescue. Emergency nurses often need to deal with patients with critical and severe diseases or with complex diseases, which may lead to rapid working rhythms and high mental tension. Therefore, nurses in the emergency department may be easier to suffer from poor sleep and the influencing factors of emergency nurses’ sleep may be different from other groups of people.

Stress, one’s response to environmental stressors, has been shown to be a hazard for many chronic diseases [28]. In the healthcare industry, stress is pervasive in the workplace [29, 30]. Nurses, especially emergency nurses, experience high mental stress from their work due to the fact of fast work pace and many emergencies [31]. Therefore, stress might contribute largely to the development of poor sleep among emergency nurses.

In Mainland China, the emergency department is the first-line place to treat critical, serious and urgent patients and also the basic component of a whole city emergency network. But considering the shortage of emergency nurses, population aging, the large population base and patients’ inclination of going to large hospitals when face emergencies [31, 32], the workload of emergency nurses might be very heavy, especially in general hospitals, which would extend the working hours or speed up the rhythms of work and may make nurses suffer more from sleep problems. And now little information is available about the current sleep status of emergency nurses in Mainland China, although previous studies conducted in 2015 reported high prevalence of poor sleep in the emergency department in China [23, 24]. However, many measures have been taken to improve the working environment of nurses and the number of registered nurses in China has been rising rapidly in the past several years, from 3.24 million (2.36 nurses per 1thousand people) at the end of 2015 [33] to over 4 million (over three nurses per 1 thousand people) by the end of 2018 [34]. What’s more, those previous studies [23, 24] only included nurses of one-level hospital and many factors such as workload, especially workload at night, which might influence sleep quality, were not collected and evaluated. This study aimed to evaluate the sleep quality of emergency nurses working in public hospitals in Shandong, China, compare the difference on sleep quality among emergency nurses in primary hospitals, secondary hospitals and tertiary hospitals, and to explore its influencing factors, including workload, occupational, psychosocial and individual factors, so as to provide information for scientific management, improvement of physical and mental health level, and improvement of quality and efficiency of emergency nursing work.

## Methods

### Participants

A cross-sectional study was implemented in Shandong, China from June to December in 2019. As there were no more than 20,000 emergency nurses in Shandong, China and we set type 1 error as 0.05 and the allowable error as 0.02, given an estimated 50% prevalence rate of poor sleep based on previous studies [23, 24], the required sample size needed to be at least 2144. After considering a non-response rate of 30%, the sample size would be 3063. Selecting 3063 emergency nurses distributed in various districts by simple random sampling, systematic random sampling or stratified sampling might be time-consuming and not economic. Thus, we preferred random cluster sampling method with the help of local health committees. As the sampling error of random cluster sampling is relative bigger than other random sampling methods, we enlarged the sample size and decided to select five clusters from among all the 17 city emergency command systems in Shandong, China considering there being about 1 thousand subjects in each city emergency command system. First, of the 17 city emergency command systems in Shandong province, five were selected randomly. The selected five city emergency command systems consisted of 224 public hospital emergency departments, 14 of which were from tertiary hospitals, 43 from secondary hospitals and 167 from primary hospitals. Then, of the 224 public hospital emergency departments, all the nurses who had been employed in the emergency outpatient department for at least 12 months were invited to participate in the study. Exclusion criteria were as follows: pregnancy, diseases such as chronic pain, hyperthyroidism, restless leg syndrome, myocardial infarction, tumor, etc., taking medicines and family history of sleep disorders. From June to December in 2019, a total of 4856 emergency nurses completed our questionnaire. Considering data collection efficiency and availability, 606 nurses failed to participate mainly due to long leaves for maternity leave, disease and personal affairs. And 126 questionnaires were discarded due to incompletely or incorrectly filling. Finally, 4730 eligible questionnaires were analyzed. Therefore, the effective response rate of the study was 86.6%.

The Ethics Committee of Shouguang People’s Hospital and Health Commission of Weifang City (wfwsjk_2019_188) approved the study. All the nurses participated in the study were voluntary and gave written informed consent.

### Survey questionnaire

A paper-based self-administered questionnaire was developed, based on advice of epidemiologists and occupational health experts, field observation, interviews with emergency nurses and established questionnaires [35–39]. The questionnaire was piloted in 32 emergency nurses and revised. And the final questionnaire comprised four sections.

Section one dealt with individual information including sex, age, body mass index (BMI, body/height squared), educational level, marital status, children in household, smoking, alcohol/tea consumption, exercise in leisure time, income and professional rank (senior/deputy senior, intermediate, primary and registered).

Section two dealt with work characteristics including hospital level (primary, secondary, tertiary), work years, number of patients in the charge of in the daytime, whether taking shift work (Yes/No), number of night shifts per month, number of patients in the charge of at night and employment status (permanent vs. temporary/contract).

Sleep quality was assessed in section three, using the Pittsburgh Sleep Quality Index (PSQI) scoring system [35] which has been translated into Chinese language and widely used in the world for the investigation, evaluation and research of sleep quality and the evaluation of the correlation between sleep quality and physical and mental health [36]. The PSQI is a self-rated scale assessing sleep quality and sleep disturbances over 1 month interval [35]. Nineteen items makes up seven component scores which are summed for one global score (0–21) with higher scores indicating worse sleep quality [35]. Generally, a global score of > 5 could be thought as poor sleep or sleep disturbance and > 8 severe sleep problem [35, 36].

The forth section dealt with occupational stress evaluated by Job Content Questionnaire (JCQ) based on the job demand-control-support model, which has been translated into Chinese language and been validated in the Chinese people [37–39]. In our study, 25 items of the original 49-item JCQ were adopted and consisted of four components: workplace social support including coworker support and supervisor support, job control including skill discretion and decision-making authority, psychological job demand, and job insecurity. The Chinese version of JCQ was widely used in China and more details could be found at our previous study [31]. The Cronbach’s alpha of our final questionnaire was 0.89 for work characteristics, 0.85 for sleep quality and 0.65 to 0.93 for components of occupational stress.

### Data analysis

Statistical analysis was conducted on SPSS version 25.0. The differences were first tested by bivariate analysis such as one way analysis of variance (for continuous variables), chi-square test (for categorical variables), or rank sum test (if not suitable for one way analysis of variance or chi-square test). Every potential influencing factor for poor sleep was individually entered into a logistic regression analysis with poor sleep as dependent variable (PSQI> 5) and odds ratio (OR) with 95% confidence interval (C.I.) was calculated. Then multivariate logistic regression analysis was used to quantify the association of potential influencing factors, including workload, occupational, psychosocial and individual factors, with poor sleep. Variables selected at the level of 0.25 in bivariate logistic regression analysis were entered into the final multivariate logistic regression model with poor sleep (PSQI > 5) as dependent variable. The statistics for variable entry and removal were set at *P* < 0.05 and *P* < 0.01, respectively. All tests were conducted at the 0.05 level of statistical significance.

## Results

Of the 4730 emergency nurses, 3349 were from primary hospitals, 1044 from secondary hospitals and 337 from tertiary hospitals. The average age of the 4730 emergency nurses was 35.3 ± 6.8 years old, including 4185 female nurses and 545 male nurses. All nurses had at least 12 years of education. The average PSQI score was 8.2 ± 3.9, including 3114 (65.8%) nurses with a PSQI > 5 and 2905 (61.4%) nurses with a PSQI > 8.

The emergency nurses in tertiary hospitals were younger than primary hospitals and secondary hospitals and had less years of service in the emergency department. The emergency nurses in tertiary hospital were in charge of more patients at night and in the daytime than those in primary hospitals and in secondary hospitals and experienced more work hours per week. The PSQI score of emergency nurses in tertiary hospitals was 11.8 ± 4.3, statistically higher than those in primary hospitals (9.5 ± 3.9) and in secondary hospitals (7.4 ± 3.5). The prevalence of poor sleep (PSQI > 5) and severe sleep problem (PSQI> 8) among emergency nurses was 76.3 and 68.8%, respectively, in tertiary hospitals, both statistically higher than in primary hospitals (63.7, 59.7%) and secondary hospitals (69.4, 64.7%). For more details, see to Table 1.

**Table 1:** Characteristics and sleep quality of nurses in the emergency department in public hospitals in Shandong, China

	Total	In primary hospitals	In secondary hospitals	In tertiary hospitals	*P*
Number of subjects	4730	3349	1044	337	
Age	35.3 ± 6.8	35.9 ± 6.5	34.6 ± 7.1	32.5 ± 7.4	< 0.001
Sex					0.082
Male	545 (11.5%)	365 (10.9%)	132 (12.6%)	48 (14.2%)	
Female	4185 (88.5%)	2984 (89.1%)	912 (87.4%)	289 (85.8%)	
Marital status					0.560
Never married	677 (14.3%)	472 (14.1%)	153 (14.7%)	52 (15.4%)	
Married/cohabiting	3427 (72.5%)	2446 (73.0%)	748 (71.6%)	233 (69.1%)	
Divorced/separated/widowed	626 (13.2%)	431 (12.9%)	143 (13.7%)	52 (15.4%)	
Tea/coffee consumption					0.975
Never/almost never	2091 (44.2%)	1475 (44.0%)	462 (44.3%)	154 (45.7%)	
Sometimes	1088 (23.0%)	779 (23.3%)	238 (22.8%)	71 (21.1%)	
Often	886 (18.7%)	639 (19.1%)	188 (18.0%)	59 (17.5%)	
Daily/almost daily	665 (14.1%)	456 (13.6%)	156 (14.9%)	53 (15.7%)	
BMI					0.115
< 18.5	373 (7.9%)	269 (8.0%)	79 (7.6%)	25 (7.4%)	
18.5 ~ 23.9	2413 (51.0%)	1668 (49.8%)	551 (52.8%)	194 (57.6%)	
24.0 ~ 27.9	1168 (24.7%)	851 (25.4%)	249 (23.9%)	68 (20.2%)	
≥ 28.0	776 (16.4%)	561 (16.8%)	165 (15.8%)	50 (14.8%)	
Exercise in leisure time					0.007
Never/almost never	2677 (56.6%)	1852 (55.3%)	615 (58.9%)	210 (62.3%)	
Sometimes	1194 (25.2%)	863 (25.8%)	253 (24.2%)	78 (23.1%)	
Often	859 (18.2%)	634 (18.9%)	176 (16.9%)	49 (14.5%)	
Personal income per month					0.001
< Ұ4000	1393 (29.5%)	1026 (30.6%)	289 (27.7%)	78 (23.1%)	
Ұ4000-	1442 (30.5%)	1029 (30.7%)	313 (30.0%)	100 (29.7%)	
Ұ6000-	1259 (26.6%)	864 (25.8%)	293 (28.1%)	102 (30.3%)	
Ұ8000-	439 (9.3%)	298 (8.9%)	102 (9.8%)	39 (11.6%)	
Ұ10,000-	197 (4.2%)	132 (3.9%)	47 (4.5%)	18 (5.3%)	
Smoking					0.975
Never smoked	4077 (86.2%)	2893 (86.4%)	896 (85.8%)	288 (85.5%)	
Ex-smoker	332 (7.0%)	231 (6.9%)	75 (7.2%)	26 (7.7%)	
Current smoker	321 (6.8%)	225 (6.7%)	73 (7.0%)	23 (6.8%)	
Educational level					< 0.001
Lower than junior college	1854 (39.2%)	1352 (40.4%)	389 (37.3%)	113 (33.5%)	
Junior college	2297 (48.6%)	1633 (48.8%)	512 (49.0%)	152 (45.1%)	
Bachelor	506 (10.7%)	329 (9.8%)	121 (11.6%)	56 (16.6%)	
Master or above	73 (1.5%)	35 (1.0%)	22 (2.1%)	16 (4.7%)	
Children in household					< 0.001
=0	441 (9.3%)	276 (8.2%)	112 (10.7%)	53 (15.7%)	
= 1	3139 (66.4%)	2194 (65.5%)	719 (68.9%)	226 (67.1%)	
= 2	1120 (23.7%)	856 (25.6%)	208 (19.9%)	56 (16.6%)	
> 2	30 (0.6%)	23 (0.7%)	5 (0.5%)	2 (0.6%)	
Professional rank					< 0.001
Registered	1294 (27.4%)	952 (28.4%)	276 (26.4%)	66 (19.6%)	
Primary	2450 (51.8%)	1856 (55.4%)	459 (44.0%)	135 (40.1%)	
Intermediate	720 (15.2%)	382 (11.4%)	235 (22.5%)	103 (30.6%)	
Senior/deputy senior	266 (5.6%)	159 (4.7%)	74 (7.1%)	33 (9.8%)	
Years of service	11.2 ± 6.5	12.1 ± 5.8	9.4 ± 6.7	7.1 ± 8.7	< 0.001
Night shift frequency per month					0.006
Never	515 (10.9%)	359 (10.7%)	121 (11.6%)	35 (10.4%)	
1–3	1179 (24.9%)	856 (25.6%)	246 (23.6%)	77 (22.8%)	
4–6	1799 (38.0%)	1325 (39.6%)	365 (35.0%)	109 (32.3%)	
≥ 7	1237 (26.2%)	809 (24.2%)	312 (29.9%)	116 (34.4%)	
Workload					
Work hours per week					< 0.001
< 40	1331 (28.1%)	1021 (30.5%)	249 (23.9%)	61 (18.1%)	
40–45	2157 (45.6%)	1530 (45.7%)	476 (45.6%)	151 (44.8%)	
> 45	1242 (26.3%)	798 (23.8%)	319 (30.6%)	125 (37.1%)	
Number of patients in the charge of at night					< 0.001
< 30	996 (21.1%)	765 (22.8%)	186 (17.8%)	45 (13.4%)	
30–60	2028 (42.9%)	1405 (42.0%)	466 (44.6%)	157 (46.6%)	
> 60	1706 (36.1%)	1179 (35.2%)	392 (37.5%)	135 (40.1%)	
Number of patients in the charge of in the daytime					< 0.001
< 30	1325 (28.0%)	1009 (30.1%)	253 (24.2%)	63 (18.7%)	
30–60	1753 (37.1%)	1211 (36.2%)	405 (38.8%)	137 (40.7%)	
> 60	1652 (34.9%)	1129 (33.7%)	386 (37.0%)	137 (40.7%)	
Employment status					0.101
Permanent	2727 (57.7%)	1961 (58.6%)	586 (56.1%)	180 (53.4%)	
Temporary/contract	2003 (42.3%)	1388 (41.4%)	458 (43.9%)	157 (46.6%)	
Sleep quality					
PSQI	8.2 ± 3.9	7.4 ± 3.5	9.5 ± 3.9	11.8 ± 4.3	< 0.001
PSQI> 5	3114 (65.8%)	2132 (63.7%)	725 (69.4%)	257 (76.3%)	< 0.001
PSQI> 8	2905 (61.4%)	1998 (59.7%)	675 (64.7%)	232 (68.8%)	< 0.001

After bivariate logistic regression analysis between poor sleepers (PSQI> 5) and good sleepers (PSQI≤5), multivariate logistic regression analysis revealed that poor sleep quality was independently positively associated with hospital level (tertiary vs. primary, secondary vs. primary), female sex, more work hours per week (> 45 vs. < 40, 40–45 vs. < 40), higher number of patients in the charge of at night (> 60 vs. < 30, 30–60 vs. < 30), higher nigh shift frequency per month (4–6 vs. never, ≥7 vs. never), higher psychological job demand and higher job insecurity, and was negatively associated with more exercise in leisure time (often vs. never/almost never, sometimes vs. never/almost never), higher workplace social support and higher job control. For more details, see to Table 2.

**Table 2:** Bivariate and multivariate logistic regression analysis of poor sleep in emergency nurses

Factor	Bivariate logistic analysis		Multivariate logistic analysis	
	*P*	Crude OR (95% C.I.)	*P*	Adjusted OR (95% C.I.)
Hospital level				
Primary hospital	Reference		Reference	
Secondary hospital	0.001	1.297 (1.117–1.506)	0.008	1.309 (1.128–1.519)
Tertiary hospital	< 0.001	1.834 (1.413–2.380)	< 0.001	1.852 (1.427–2.403)
Sex (0 = male, 1 = female)	< 0.001	2.908 (2.425–3.487)	0.039	2.048 (1.036–4.051)
Exercise in leisure time				
Never/almost never	Reference		Reference	
Sometimes	< 0.001	0.410 (0.355–0.473)	0.041	0.448 (0.209–0.957)
Often	< 0.001	0.327 (0.278–0.383)	< 0.001	0.352 (0.169–0.735)
Workload				
Work hours per week				
< 40	Reference		Reference	
40–45	< 0.001	2.337 (2.029–2.693)	0.042	1.908 (1.021–3.565)
> 45	< 0.001	3.104 (2.623–3.672)	< 0.001	2.517 (1.270–4.988)
Number of patients in the charge of at night				
< 30	Reference		Reference	
30–60	< 0.001	2.560 (2.191–2.991)	0.012	2.201 (1.102–4.397)
> 60	< 0.001	8.062 (6.721–9.670)	< 0.001	6.080 (3.190–11.586)
Night shift frequency per month				
Never	Reference		Reference	
1–3	< 0.001	2.279 (1.839–2.824)	0.146	2.018 (0.918–4.437)
4–6	< 0.001	4.339 (3.527–5.339)	< 0.001	3.717 (1.807–7.647)
≥ 7	< 0.001	9.050 (7.162–11.438)	< 0.001	7.156 (3.357–15.256)
Occupational stress				
Psychological job demand	0.003	2.257 (1.086–4.688)	0.018	2.168 (1.062–4.426)
Job control	< 0.001	0.425 (0.195–0.927)	< 0.001	0.451 (0.212–0.961)
Workplace social support	< 0.001	0.348 (0.158–0.765)	< 0.001	0.378 (0.180–0.794)
Job insecurity	0.011	2.447 (1.054–5.685)	0.022	2.408 (1.045–5.551)

## Discussion

In the study, we found that the mean PSQI score of nurses in the emergency department in public hospitals in Shandong, China was 8.2 ± 3.9, most highly found in tertiary-hospital emergency nurses (11.8 ± 4.3), followed by secondary-hospital emergency nurses (9.5 ± 3.9) and primary-hospital emergency nurses(7.4 ± 3.5). The prevalence of poor sleep quality (PSQI> 5) and severe sleep problems (PSQI> 8) of emergency nurses was 76.3 and 68.8%, respectively, in tertiary-hospital emergency nurses, both statistically higher than secondary-hospital emergency nurses (69.4 and 64.7%) and primary-hospital emergency nurses (63.7 and 59.7%). The poor sleep quality among emergency nurses was associated with hospital level (tertiary vs. primary, secondary vs. primary), workload (work hour per week, number of patients in the charge of at night), night shift work and work stressors (low workplace social support, low job control, high psychological job demand, high job insecurity), in addition to individual factors (sex and exercise in leisure time). More exercise in leisure time was found to be negatively associated with poor sleep among emergency nurses.

To our knowledge, this may be the first large-scale multi-center study assessing sleep quality of emergency nurses working in public hospitals including primary, secondary and tertiary hospitals in China and its influencing factors, by applying standardized and well-validated instruments to diagnose sleep quality and measure occupational stress. The emergency nurses participated in the study were sampled from among all the 17 city emergency command systems in Shandong, China and the response rate of 86.6% was acceptable. The limitations of the study include the cross-sectional design and self-report measures, not use objective tests. Therefore, no solid causal directions could be made from the cross-sectional study. A cohort study might be needed in the future. As this study collected data on sleep among emergency nurses in public hospitals only, the results might not be generalized to other population, such as emergency nurses in private hospitals.

In our study, the prevalence of poor sleep and severe sleep problem was 65.8 and 61.4%, respectively, in public-hospital emergency nurses, higher than other hospital workers [22–24, 40, 41]. Studies found the prevalence of poor sleep or sleep problem was 43.1% (PSQI> 5) in healthcare workers in Tehran [41], 48.6% (PSQI≥5) in nurses in Saudi Arabia [40], 57% (PSQI≥5) in nurses in Taiwan [26], 58.3%(PSQI > 5) in nurses in Nigeria [22], 63.9% (PSQI ≥5) and 54.8% (PSQI ≥8) in nurses in China [23, 24]. In systematic reviews and meta-analyses, the pooled prevalence of poor sleep or sleep disturbances was reported to be 51% (95% CI 42–60%) in 3722 police officers [42], 39.2% (95% CI 36.0–42.7%) among 31,749 Chinese healthcare professionals [43], lower than our results. Besides, we also found that the prevalence of poor sleep and severe sleep problems among emergency nurses in tertiary hospitals was statistically higher than those in secondary and primary hospitals. Therefore, emergency nurses in public hospitals, especially in tertiary hospitals, are prone to suffer from poor sleep than other health care workers or nurses in other departments and its influencing factors need to be identified.

Studies [44, 45] have reported that hospital nurses were highly stressed. We found that high job stress (low workplace social support and high psychological job demand) was independently associated with poor sleep, similar to other studies [46, 47]. The unique environment in the emergency department requires quick decision making, specialized and demanding nursing and in-time measures taking. At the same time, emergency nurses had to not only perform accurate clinical assessment but also remain vigilant in order to fast respond to changes of patient’s unstable conditions. Our study also showed that workplace social support was negatively correlated with poor sleep. Workplace social support including coworker support and supervisor support is considered to help buffer the negative effects of high job stress (high job demand and low job control) on health [48]. Enough workplace social support has been shown to be associated with decreased risk of psychiatric disorders [49] and low work absence due to psychiatric disorders [50]. Therefore, measures such as conflict resolving and supporting systems might be suggested in the emergency department in public hospitals in China.

Emergency nurses with more night shifts per month (4–6 vs. never, ≥7 vs. never) suffered from poor sleep more easily, consistent with other studies [16, 51–54]. Further, the OR (7.156, 95% C.I. = 3.357–15.256) for ≥7 number of night shift per month was higher, compared with other factors. At present, patients tend to go to general hospitals for their first doctor visit and emergency nurses were in shortage, especially in tertiary hospitals. We found that many emergency nurses had to take at least 7 night shifts per month, especially in tertiary hospitals (Table 1). In addition, when shift nurses sleep during the day, the sleep quality was difficult to guarantee because of noisy daytime environment, poor sleep environment, and daytime light which could make melatonin secretion reduced. Therefore, it seems to be important to control the number of night shift for emergency nurses and provide a good place for night shift workers to sleep.

In this study, high number of patients in the charge of at night and long work hours per week were associated with emergency nurses’ poor sleep. Number of patients in the charge of at night and work hours per week could be taken as signs of workload, and increased workload has been proved to be a hazard for fatigue or stress [55]. Both demanding work and long work hours has been demonstrated to be associated with sleep difficulties in a longitudinal French cohort study [56]. The mechanism between workload and poor sleep remains unclear, possibly because the more patients they were in charge of, the more stress or fatigue they would experience. What’s more, the OR of the number of patients in the charge of > 60 at night (6.080, 3.190–11.586) was greater (Table 2), which might mean that heavy workload at night contributes largely to the development of poor sleep, in addition to night shift frequency per month of ≥7 (Table 2). In addition, we found often exercise in leisure time was negatively associated with poor sleep, consistent with previous studies [57, 58]. A systematic review suggested exercise could promote increased sleep duration and efficiency regardless of the intensity and mode of activity [57]. In another systematic review and meta-analysis, exercise was shown to improve sleep quality without notable adverse effects [58]. Maybe exercise could relieve stress and then improve sleep quality, although the mechanism has not been illuminated, exercise was recommended for emergency nurses.

## Conclusions

Attention should be given to the high prevalence of poor sleep of nurses in the emergency department in public hospitals in China. Factors of many night shift taking, high workload at night and long work hours per week were significantly associated with emergency nurses’ poor sleep, in addition to individual factors such as sex and exercise. Exercise and decreasing occupational stress might be considered when exploring preventive measures for poor sleep in emergency nurses.

## Data Availability

Data will not be shared publicly but could be accessed from the corresponding author on reasonable request.

## Abbreviations

PSQI: Pittsburgh Sleep Quality Index
JCQ: Job Content Questionnaire
OR: Odds ratio
C.I: Confidence interval
